# Advanced Interpretation of Field Cycling NMR Relaxometry Dispersion Profiles From Hard and Soft Materials

**DOI:** 10.1002/mrc.70089

**Published:** 2026-03-05

**Authors:** David A. Faux, Rémi Kogon

**Affiliations:** ^1^ Department of Physics University of Surrey Guildford UK

**Keywords:** 3‐Tau model, fast field‐cycling NMR, NMR relaxometry, surface chemistry

## Abstract

A fast field‐cycling NMR (FFC NMR) experiment measures the longitudinal (spin–lattice) relaxation rate as a function of applied magnetic field to yield a relaxation rate dispersion curve, $R_{1}\left(f\right),$ where f is the proton Larmor frequency. The $R_{1}\left(f\right)$ dispersions, or NMRD profiles, are exquisitely sensitive to the relative dynamics of proton spins across timescales in the range 10^−9^–10^−4^ s. These timescales span the translational and rotational dynamics of proton‐bearing fluids to dynamics at the surfaces of solids, soft material and macromolecules. FFC NMR is useful for studying fluid‐filled rocks and soils, porous silica and cementitious material, polymer systems, foodstuffs, protein systems, biological tissues and biofluids. The NMRD profiles are rich with information, but interpretation is challenging. A parametrized relaxometry model must generate an NMRD profile $R_{1}\left(f\right)$ that can be fit to experimental data across three to four orders of magnitude of frequency. The 3‐Tau model has emerged as a model capable of fitting NMRD profiles from a broad range of material types yielding physically meaningful parameters. This review article demonstrates power of the FFC NMR experiment interpreted using the 3‐Tau model to reveal properties of hydrated hard and soft material.

## Graphical abstract

This review demonstrates that NMR dispersion profiles from fast field cycling NMR relaxometry can be interpreted with the 3‐Tau model for a broad spectrum of soft and hard materials. Meaningful physical quantities provide insight into surface chemistry, bound water density, pore size, aqueous or solid iron (III) density and the free water diffusion coefficient. We show that changes in the physical quantities can reveal curing rates and biomarkers for tumours and sickle cell anaemia.

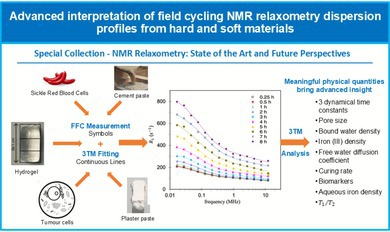

## 1 Introduction

A nuclear magnetic resonance (NMR) relaxometry experiment normally measures the longitudinal (spin–lattice) relaxation time $T_{1}$ or the transverse (spin–spin) relaxation time $T_{2}$ at a fixed applied magnetic field. Fast field cycling (FFC) NMR relaxometry is a specialized technique that changes the magnetic field to measure the relaxation rate $R_{1}=T_{1}^{-1}$ as a function of field strength across the proton Larmor frequency range typically f=0.01−40 MHz. The NMR dispersion (NMRD) profiles, $R_{1}\left(f\right)$, are rich with information about the dynamic environments of proton‐bearing fluids with applications to a broad range of soft and hard material [1, 2, 3, 4].

The dynamical correlation times accessible to a FFC NMR experiment are typically in the range 0.01–10 0μs and are associated with the relative rotational and/or translational motion of pairs of spins. Furthermore, dynamical correlation times shorter than those directly accessible, typically 0.01–10 0 ns, can be obtained if the asymptotic high frequency limit of an NMRD profile, $R_{1}\left(f\to \infty \right)$, can be estimated. However, it is not just proton dynamics that define $R_{1}\left(f\right)$. The shape and magnitude of the NMRD profiles also contain information about the presence (or otherwise) of paramagnetic ions, the spin density of bound fluid and pore size information.

In view of the information captured by NMRD profiles, it might be surprising that FFC NMR is not more widely used. One reason is that the FFC NMR experiment requires specialist equipment resulting in limited access to instruments in Europe and a similar shortage in North America and elsewhere. Further, most machines are dedicated to a specific material class. However, the most significant issue is the difficulty in extracting the rich information captured by the NMRD profiles from complex systems.

This article is organized as follows. In Section 2, the physical principles that underpin dipolar relaxation models are described. It would be impossible to review all relaxation models, so we limit the discussion to classes of models most often used for the interpretation of NMRD profiles. Their strengths, weaknesses and limitations are discussed. In addition, the Hwang–Freed (HF) relaxation model for proton‐bearing bulk or ionic liquids is described [5, 6]. Relaxation models for aqueous paramagnetic ions [7] and quadrupolar relaxation [8] can supplement general relaxation models and are summarized where used. We review the science underpinning the 3‐Tau model [9, 10] (3TM) before using the 3TM to analyse NMRD profiles from hydrated cement, plaster paste, hydrogel, tumorous tissue and haemoglobin in Section 3. The 3TM is state‐of‐the‐art and chosen because it is a single model that can be applied flexibly to hard material, soft material and macromolecular systems to supply a broad range of physically meaningful material parameters.

## 2 Interpreting NMRD Profiles

### 2.1 Model Requirements

A parametrized relaxometry model must generate an NMRD profile that can be fit to the experimental data. An ideal and complete model contains three elements: It must describe the dynamics of the water proton spins moving relative to other protons and/or paramagnetic ions, account for the distance and angular dependence of the spin‐pair interactions and must appropriately account for the system geometry insofar as it impacts the spin‐pair dynamics.

The master equation for dipolar relaxation obtained from Abragam [11] and reproduced as Equation (5) in reference [9] is given by

(1) $$G\left(t\right)=\frac{4\pi }{5}\int_{{\mathbb{R}}^{3}}\int_{{\mathbb{R}}_{0}^{3}}\left[\sum_{M=-2}^{2}\frac{Y_{2M}\left(r_{0}\right)Y_{2M}^{*}\left(r\right)}{r_{0}^{3}r^{3}}\right]P\left(r,t\cap r_{0}\right)\;d^{3}r_{0}\;d^{3}r.$$

Equation (1) contains the three key components necessary to determine the dipolar correlation function $G\left(t\right)$. First, the term in square brackets captures the dipolar interactions where Y are spherical harmonic functions of degree 2. Second, the conditional probability density function $P\left(r,t\cap r_{0}\right)$ describes the spin pair dynamics and gives the probability distribution for pairs of spins separated by $r_{0}$ at t=0 *and* by r at time t. Finally, the spatial limits on the integrals capture the spin‐pair geometry for a specific system.

In practice, the conditional probability density function $P\left(r,t\cap r_{0}\right)$ is decomposed into its rotational and translational components. That is, $P\left(r,t\cap r_{0}\right)$ is the diffusion equation Green's function that describes the time‐dependent change in distance between Spin 1 and Spin 2 obtained as a solution to Fick's second law. $P\left(r,t\cap r_{0}\right)$ substituted into Equation (1) produces the dipolar correlation function $G\left(t\right)$ for translational dynamics. Similarly, $P\left(\theta ,t\cap \theta_{0}\right)$ describes the time‐dependent change in the angle of the vectors r and $r_{0}$ with respect to a fixed axis and yields $G\left(t\right)$ for rotational dynamics.

The Fourier transform of $G\left(t\right)$ yields the spectral density function $J\left(\omega \right)$, where ω=2πf, and hence the frequency‐dependent longitudinal (spin–lattice) and transverse (spin–spin) relaxation rates $R_{1}\left(\omega \right)$ and $R_{2}\left(\omega \right)$, respectively [11]. Most models bypass Equation (1) to make simplifying assumptions about $G\left(t\right)$ or $J\left(\omega \right)$ to formulate a parametrized expression that can be fit to the NMRD profiles. As far as we are aware, only the 3TM and the HF model described in Section 2.7 solves Equation (1) explicitly for specific geometry and then only consider relative *translational* spin dynamics using the diffusion equation Green's function, neglecting the rotation of spin pairs. Other models make simplifying assumptions for $G\left(t\right)$ or $J\left(\omega \right)$, to formulate a parametrized expression that can be fit to the NMRD profiles. The addition of a frequency‐independent relaxation rate offset and a quadrupolar contribution may also be necessary to secure satisfactory fits to NMRD profiles.

A selection of widely used models is presented in Sections 2.2, 2.7, and the 3TM is reviewed in Section 2.8.

### 2.2 The BPP and SBM Models

The first successful theoretical description of the NMRD profiles for a bulk liquid was provided by Bloembergen, Purcell and Pound [12], commonly referred to as BPP theory, for a bulk liquid (glycerol) in which $G\left(t\right)$ decays exponentially at a rate determined by a characteristic time constant. The Fourier transform of $G\left(t\right)$ supplies a convenient analytic expression for spin‐bearing liquids for $J\left(\omega \right)$ and hence the relaxation rate, albeit that the exponential assumption for $G\left(t\right)$ does not work particularly well for glycerol.

Solomon, Bloembergen, Morgan (SBM) and others [13, 14, 15, 16, 17] adopted a simplified model of the dipolar contribution to NMRD profiles from liquids containing paramagnetic ions by considering only rotational motions. These authors fixed the spin–spin distance and treated the rotational dipolar correlation function $G\left(t\right)$ as a single exponential function. The SBM model produced good matches to inflection features of NMRD profiles and has since been the first port of call for the interpretation of experimental dispersions from, for example, liquids that contain paramagnetic ions.

### 2.3 The Korb Models

Jean‐Pierre Korb has made significant contributions to interpreting NMRD profiles from porous solids where the NMRD profiles are dominated by the interaction of the electronic spins of paramagnetic ions with nuclear spins of mobile protons, normally water. One widely used Korb model assumes that the ^1^H spins of water in a single two‐dimensional (2D) layer at solid surfaces diffuse within the layer making repeat encounters with paramagnetic impurities also located in the 2D surface layer [1, 18, 19, 20, 21, 22, 23, 24, 25].

The dipolar correlation function

(2) $$G\left(t\right)\propto t^{-1}\left(e^{-t/\tau_{d}}-e^{-t/\tau_{\ell }}\right)$$

is then constructed with two BPP‐like exponentially decaying functions with dynamical time constants associated with desorption ( $\tau_{d})$ and layer self‐diffusion ( $\tau_{\ell })$ multiplied by $t^{-1}$ which guarantees the correct two‐dimensional (2D) diffusive behaviour at long times. Good fits to many systems including hydrated solids such as rock and cementitious material were obtained. Korb had identified the key physics, and this model plus other variants remain widely used. Shortcomings of the model are discussed by Faux et al. [10]. Chiefly, only the desorption time constant is physically meaningful ( $\tau_{d})$, and the reduction to 2D dynamics yields unrepresentative model NMRD profiles at low frequencies when the paramagnetic spins are distributed throughout the solid material rather than just at the solid surfaces [10].

### 2.4 Multi‐Site Models

Multi‐site models typically package two or three BPP‐like expressions, each with a dynamic time constant, with other fit parameters (which may or may not be physically meaningful) to provide expressions for the relaxation rate that are fit to NMRD profiles. Multi‐site models are most used for systems of proteins and other macromolecules where alternative approaches are limited [26, 27, 28, 29, 30, 31, 32, 33, 34]. Examples of multi‐site models are the Two Sites Water Exchange Model (2SWEM) and the Three Sites Exchange Model (3SEM) [32, 33, 34]. The time constants revealed by the fitting are likely to be meaningful, and changes in the physical property of a set of samples can often be linked to a systematic change in a particular fit parameter. Shortcomings are that it is difficult to associate a dynamic time constant with a physical process, and incorrect assignations are often made if molecular dynamics simulations are not available to assess the validity of dynamical timescales.

More sophisticated multi‐site models associate well‐defined dynamical processes with each water environment and associate a suitable model for each. Examples are Kruk et al. [35] who interpreted NMRD profiles from a protein system in terms of three diffusion models: a dipolar three‐dimensional translational diffusion model, a two‐dimensional surface diffusion model and a model of surface diffusion mediated by adsorption and desorption. Chávez and Halle [36] devised an exchange‐mediated orientational randomization (EMOR) model [36, 37] for proton relaxation in gels including interactions between fixed and mobile protons. Excellent examples of a multi‐site model for a non‐aqueous system come from Kruse et al. [38, 39] who describe the NMRD profiles from ionic liquids with ^1^H and ^19^F spins using a combination of rotational, BPP and Hwang Freed relaxation models.

### 2.5 Power Law Models

A dynamical model may lead to power law expression for $J\left(\omega \right)$, and hence $R_{1}\left(\omega \right)$, in the limit ωτ→0 or ωτ→∞. Limiting power law forms for translational motion for ωτ≫1, where τ is a correlation time, was advanced by Ayant et al. [6] and later extensively explored by Sholl [40] for a variety of diffusion models. Halle [41] supplied power law solutions for the EMOR model, and Korb [1] also usefully exploited limiting power law expressions. Power laws for $R_{1}\left(\omega \right)$ in the limit ωτ→0 can be matched to NMRD profiles at the lowest frequencies to identify candidate dynamical processes.

### 2.6 Model‐Free Methods

Model‐free software allows the user to select from a range of functions to achieve fits to NMRD curves [42, 43, 44, 45, 46, 47]. These can be useful, for example, to model quadrupolar resonance peaks. The model‐free software virtually guarantees a good fit and it is sometimes possible to associate the change in a fit parameter with the change of an external property such as temperature. The readily available software makes the fitting process straightforward. A shortcoming is that minimal physical understanding of the system is gained because parameters in general cannot be associated with a physical property. Once again, although time constants obtained from model‐free fitting may have physical meaning, identifying the physical process can be problematic. For instance, microsecond time constants are often associated with the rotation of macromolecules such as proteins [43]. A macromolecule is surrounded by water which prohibits such rapid decorrelation of angular position whereas dynamical time constants for bound water are often at microsecond timescales as we demonstrate in Section 3.

### 2.7 The HF Model

The Hwang‐Freed (HF) model is a continuum diffusion model yielding the relaxation rates for proton‐bearing bulk or ionic liquids [5, 6]. The HF model simplifies the probability density function in Equation (1) by describing the change in *magnitude* (not angle) of the spin pair vectors by the diffusion equation. Hwang and Freed imposed a hard‐wall boundary condition at a radius $d_{\mathrm{HF}}$ so that $d_{\mathrm{HF}}$ can be considered the distance of nearest approach of pairs of spins on different water molecules. Hwang and Freed used the diffusion equation Green's function to obtain the spectral density function for $J_{\mathrm{HF}}\left(\omega \right)$ which is normally expressed as an integral equation. The limiting integral equation for $J_{\mathrm{HF}}\left(\omega \right)$ presented in reference [5] was, to our knowledge, first solved by Faux et al. to yield [7]

(3) $$J_{\mathrm{HF}}\left(\omega \right)=\frac{12\pi^{2}N_{b}\tau_{f}}{d_{\mathrm{HF}}^{3}}\frac{2\sigma^{2}+5\sqrt{2}\sigma +8}{\sigma^{6}+4\sqrt{2}\sigma^{5}+16\sigma^{4}+27\sqrt{2}\sigma^{3}+81\sigma^{2}+81\sqrt{2}\sigma +81}$$

where $\sigma =\sqrt{\omega \tau_{f}}$ and $\tau_{f}$ is the diffusion correlation time for the fluid and $N_{b}$ is the fluid proton spin density.

The HF model suffers the same shortcomings as other translational diffusion models. Rotational dynamics are excluded. In water, for instance, only approximately one half of the relaxation rate is due to translational dynamics [11, 48, 49, 50, 51]. However, $d_{\mathrm{HF}}$, which normally is set to the spin–spin distance of closest approach, can be used as an adjustable parameter to ensure that the HF model supplies the correct self‐diffusion coefficient $D_{\mathrm{HF}}=d_{\mathrm{HF}}^{2}/6\tau_{f}$ for the fluid at room temperature.

In the case of pure bulk water, the HF relaxation rate $R_{1}^{\mathrm{HF}}\left(\omega \right)$ obtained from Equation (3) is frequency independent across the frequency range of an FFC NMR experiment. The HF model therefore allows an equivalent pore water self‐diffusion coefficient $D_{\mathrm{HF}}$ to be obtained from a frequency‐independent relaxation rate $R_{1}^{\text{offset}}$ obtained from fits to NMRD profiles. If the pore water dynamics is two or more orders of magnitude slower, however, $R_{1}^{\mathrm{HF}}\left(\omega \right)$ has a weak frequency dependence and it becomes necessary to specify a frequency for the estimate of the equivalent $D_{\mathrm{HF}}$.

### 2.8 The 3‐Tau Model

**FIGURE 1:**
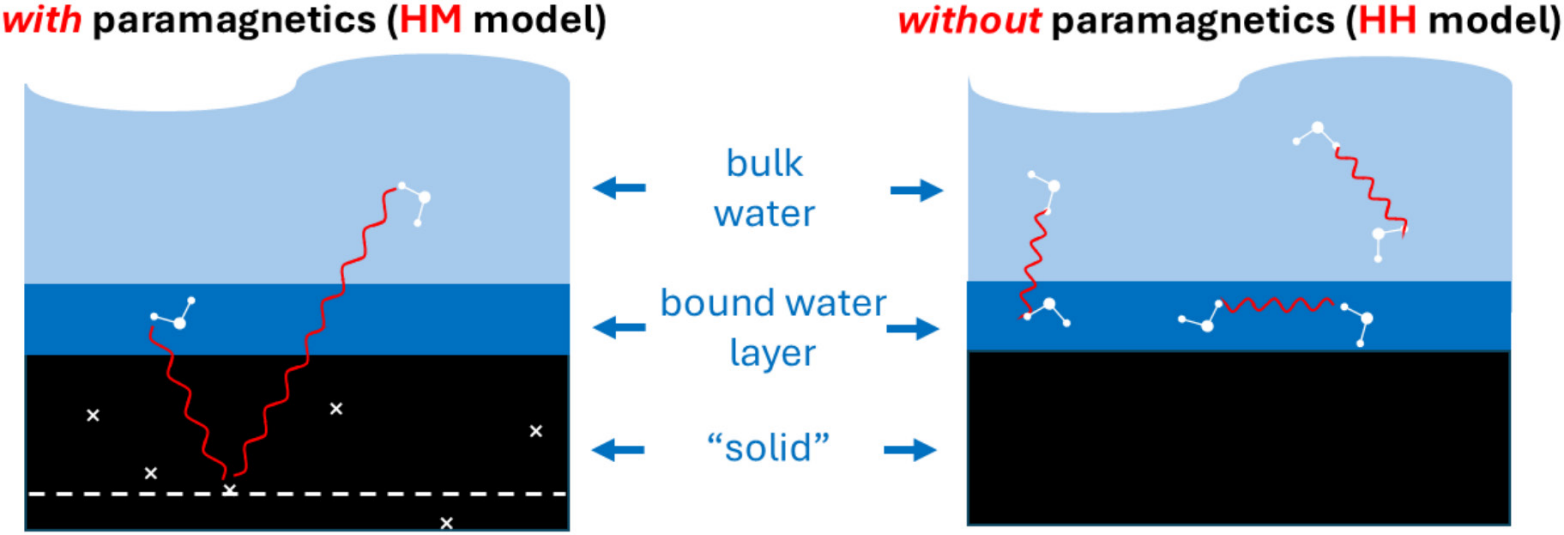
The 3‐Tau model. The single water layer is δ = 0.27 nm thick and is bound to the solid. The remaining water is bulk water. The left image illustrates the HM model showing interactions between surface and bulk water with a density of paramagnetic ions modelled as a layer (dashed white line) in the solid placed 2 δ below the surface. The image at right illustrates the HH model which includes layer–layer, bulk–bulk and bulk–layer interactions. The solid proton (SP) model is the same as the HM model except that the dashed white line represents the proton density in the solid.

The 3TM was originally developed by Faux et al. to describe NMRD profiles for hard materials with relaxation rates dominated by paramagnetic ions [9, 10, 52]. It incorporates the most appealing features of the Korb models and has subsequently been adapted to cater for materials free from paramagnetic ions. The 3TM model now considers ‘solid’ material to be hard or soft material, or macromolecules. The fluid is a proton bearing fluid and exists in two distinct environments as illustrated in Figure 1 for water. A single layer of slow‐moving water bound to the solid has thickness δ=0.27 nm representing the average distance between nearest neighbour oxygen atoms in water. The bulk water, also referred to as pore water or free water, is represented by the water atop the bound layer with no specified thickness.

The 3TM comprises three primary models, which may be used separately or together. These are the HM, HH and SP models. The HM model accounts for interactions between mobile ^1^H spins of water and fixed electronic spins in the solid thus,

(4) $$R_{1}^{\mathrm{HM}}={\mathrm{xR}}_{1}^{\mathrm{l\sigma}}+\left(1-x\right)R_{1}^{\mathrm{b\sigma}},$$

where σ labels paramagnetic ions, l refers to the surface layer (thickness = 0.27 nm), b represents the bulk and x is the dimensionless spin surface‐to‐volume ratio. See Table 1 for a summary of parameters. Relaxation rates $R_{1}^{\mathrm{l\sigma}}$ and $R_{1}^{\mathrm{b\sigma}}$ are proportional to the paramagnetic ion spin density in the solid, $N_{\sigma }$, represented by a fictional layer placed at a distance 2 δ below the surface.

**TABLE 1:** The possible 3TM fit parameters are presented with their physical meaning and explanatory comments.

Quantity	Description	Notes
$\tau_{l}$	Bound fluid dynamic correlation time	The bound fluid two‐dimensional self‐diffusion coefficient is $\delta^{2}/4\tau_{l}$ where δ=0.27 nm
$\tau_{d}$	Desorption correlation time	A measure of the lifetime of a surface‐bound fluid
$\tau_{b}$	Bulk fluid dynamic correlation time	The bulk fluid three‐dimensional self‐diffusion coefficient is $\delta^{2}/6\tau_{b}$ where δ=0.27 nm
x	Spin surface‐to‐volume ratio	Equal to Aδ/V if $N_{b}=N_{l}$where A and V represent the ‘solid’ surface area and fluid volume respectively
$N_{\sigma }$	Paramagnetic ion spin density	A representative density of paramagnetic ions uniformly distributed in the ‘solid’ material
$N_{b}$	Bulk fluid proton spin density	Fixed at $N_{b}=66.6$ spins/nm^3^ for pure water at room temperature
$N_{l}$	Bound proton spin density	$N_{l}$ < $N_{b}$ typically for soft materials
$N_{\mathrm{SP}}$	Solid proton spin density	The density of proton spins in the ‘solid’ material
$R_{1}^{\text{offset}}$	A frequency‐independent relaxation rate	Associated with restricted diffusion of bulk fluid
$R_{1}^{\mathrm{aq}}\left(f\right)$	Aqueous paramagnetic iron (III) density	Contributes to $R_{1}^{\mathrm{aq}}\left(f\right)$ for aqueous paramagnetic iron (III)

*Note:* The ‘solid’ refers to hard solids, soft material or macromolecules. The fit parameters used are model‐dependent and listed in Table 2.

The HM model illustrated in Figure 1 shows the two interactions that lead to relaxation rates $R_{1}^{\mathrm{l\sigma}}$ and $R_{1}^{\mathrm{b\sigma}}$. The particle–particle interaction between a single electronic spin within the layer of paramagnetic ions and a ^1^H spin at distance d scales as $d^{-6}$. See Equation (1). The interaction between the paramagnetic *layer* and a *layer* of bulk water distance d away scales as $d^{-2}$. Assuming each bulk layer has thickness δ, an elementary calculation finds that 90% of the contribution to $R_{1}^{\mathrm{b\sigma}}$ is due to the first four layers of bulk water, or about 1.1 nm. The value of $\tau_{b}$ that emerges from fits to NMRD profiles from the 3TM is therefore sensitive to the first four layers of bulk water atop the bound layer. Depending on the system, $\tau_{b}$ may or may not characterize the dynamics of all the pore water.

The HH model captures interactions between surface and bulk ^1^H spins [10]. The relaxation rate is found from

(5) $$R_{1}^{\mathrm{HH}}=x\left(R_{1}^{\mathrm{ll}}+R_{1}^{\mathrm{lb}}\right)+\left(1-x\right)\left(R_{1}^{\mathrm{bb}}+R_{1}^{\mathrm{bl}}\right),$$

where $R_{1}^{\mathrm{bl}}$ and $R_{1}^{\mathrm{ll}}$ are proportional to the bound layer spin density $N_{l}$, and similarly, $R_{1}^{\mathrm{lb}}$ and $R_{1}^{\mathrm{bb}}$ are proportional to the bulk water spin density $N_{b}$. For the HH model, 99% of the contribution to $R_{1}^{\mathrm{lb}}$ or $R_{1}^{\mathrm{bl}}$ is due to the first four layers of bulk water.

The SP model captures interactions between fixed ^1^H spins in the solid and ^1^H spins of the bound water layer and bulk water. The SP model adopts the same physical principles as the HM model except that the paramagnetic ions labelled σ are replaced by SP spins labelled H so that

(6) $$R_{1}^{\mathrm{SP}}={\mathrm{xR}}_{1}^{\mathrm{lH}}+\left(1-x\right)R_{1}^{\mathrm{bH}}$$

where relaxation rates $R_{1}^{\mathrm{lH}}$ and $R_{1}^{\mathrm{bH}}$ are proportional to the proton density $N_{\mathrm{SP}}$ in the solid. The individual model relaxation rates add to produce the 3TM frequency‐dependent relaxation rate $R_{1}^{3\mathrm{TM}}$,

(7) $$R_{1}^{3\mathrm{TM}}=R_{1}^{\mathrm{HM}}+R_{1}^{\mathrm{HH}}+R_{1}^{\mathrm{SP}}$$

For ease of viewing, the dependence on frequency f of all relaxation rates is omitted from Equations ((4), (7)). The HM, HH and SP models are listed in Table 2.

**TABLE 2:** The 3TM model can include one or more of the HM, HH and SP models.

Model label	Description	Fit parameters
HM	Interaction of paramagnetic ions in the solid with both surface bound spins and bulk spins	$\tau_{l}$ $\tau_{d}$ $\tau_{b}$ x $N_{\sigma }$
HH	Interactions between surface bound spins and bulk spins	$\tau_{l}$ $\tau_{d}$ $\tau_{b}$ x $N_{l}$
SP	Interaction of proton spins in the solid with both surface bound water and bulk spins	$\tau_{l}$ $\tau_{d}$ $\tau_{b}$ x *N*_*SP*_
Offset	A frequency‐independent relaxation rate	$R_{1}^{\text{offset}}\;$
Fe (aq)	Relaxation rate due to aqueous iron (III) in the bulk water	$N_{\mathrm{aq}}\;$

*Note:* The fit parameters used for each model are presented. The models labelled ‘Offset’ and ‘Fe (aq)’ may be added. The parameter required for each is listed.

There may be contributions to the relaxation rates additional to those from Equations ((4), (7)). A frequency‐independent relaxation rate $R_{1}^{\text{offset}}$ is often required to achieve satisfactory fitting. $R_{1}^{\text{offset}}$ is associated with the dynamics of the pore water and is approximately constant across the frequency‐range of an FFC NMR experiment. $R_{1}^{\text{offset}}$ for pure water at room temperature is only 0.29 s^−1^ [53] whereas the offset necessary to achieve satisfactory fitting can be substantially larger, sometimes more than 100 s^−1^ in cementitious material. Large $R_{1}^{\text{offset}}$ values in hydrated cement are due to the presence of aqueous ions, which inhibit diffusion and bind to water as described in Section 3.1.

Aqueous paramagnetic ions, chiefly iron (III), may also contribute to the overall relaxation rate. This contribution is labelled $R_{1}^{\mathrm{aq}}$. Measurements at different concentrations of chlorides of iron (III), manganese (II) and copper (II) were presented by Faux et al. [7] and the NMRD profiles for 1‐mmol/L samples were fit to a dynamical model allowing the contribution of aqueous paramagnetic ions to be incorporated into the fitting. A summary of the 3TM fit parameters is presented in Table 1. Table 2 lists the 3TM models with their model labels and the fit parameters employed for each.

A representative measure of pore size, h, can be obtained from the spin surface‐to‐volume ratio x for an assumed pore geometry. The 3TM assumes the bulk fluid is contained in planar pores. We refer to h as the ‘planar‐pore‐equivalent pore size’. A planar pore has two surface layers each of thickness δ=0.27 nm where δ is the average distance between nearest‐neighbour water oxygen atoms and represents the thickness of a single layer of water. Consequently, the planar pore spin surface‐to‐volume ratio is x=2δ/h if the proton spin density in the surface layers, $N_{l}$, is the same as in the bulk, $N_{b}$. See Table 1. However, if $N_{l}\neq N_{b}$ as is often the case for soft materials, then $x=2\delta N_{l}/N_{b}h$ leading to the general expression

(8) $$h=\frac{2\delta N_{l}}{xN_{b}}.$$

A different assumption on pore geometry will yield a different relationship between h and x. Consequently, the numerical values of h are less important than how h varies between samples or in time. For systems of macromolecules, such as proteins and blood, h may be considered a representative distance between macromolecules. Other quantities derived from the 3TM fitting are presented in Table 3.

**TABLE 3:** The physical quantities that may be derived from the 3TM fit parameters are listed.

Derived quantity	Description	Notes
h	A representative pore size	A representative pore size or distance between macromolecules derived from x using Equation (8)
$D_{b}$	Bulk water self‐diffusion coefficient from $\tau_{b}$	If all the bulk water was characterized by $\tau_{b}$, then $D_{b}=\delta^{2}/6\tau_{b}$ where δ=0.27 nm
$D_{l}$	Surface water 2D self‐diffusion coefficient	Given by the expression $D_{l}=\delta^{2}/4\tau_{l}$ where δ=0.27 nm
$D_{\mathrm{HF}}$	Bulk water self‐diffusion coefficient from $R_{1}^{\text{offset}}$	$R_{1}^{\text{offset}}$ can be converted to an equivalent self‐diffusion coefficient using the HF model. See Section 2.7.
τdτl	Number of surface hops before desorption	Originally defined by Korb [1]. This ratio is typically of order unity.
$R_{2}\left(f\right)$	Spin–spin or transverse relaxation rate	NMRD profiles are fit using 3TM to yield $R_{1}^{3\mathrm{TM}}\left(f\right)$. $R_{2}^{3\mathrm{TM}}\left(f\right)$ is also generated using the same fit parameters.
T1T2	Relaxation time ratio	This ratio is often known from conventional fixed field relaxometry. Can be used as a fit constraint with the interface.

*Note:* The Hwang–Freed (HF) model is discussed in Section 2.7.

The calculation of the relaxation rate contributions shown in Equations ((4), (7)) are described in references [9] and [10]. The relaxation rate contributions are pre‐calculated for 16 logarithmically spaced time values per decade for the three time constants $\left\{\tau_{l},\tau_{d},\tau_{b}\right\}$. The relaxation rate data files are read by fitting software incorporated in a user‐friendly interface [54, 55] or as MATLAB files. Taking the HM model as an example, the 3TM software allows the user to select ranges of the three time constants plus user‐chosen range of discrete values of the spin surface‐to‐volume ratio x and $N_{\sigma }$. $R_{1}^{3\mathrm{TM}}\left(f\right)$ is then calculated for all combinations of discrete values within the selected ranges of $\left\{\tau_{l},\tau_{d},\tau_{b},x,N_{\sigma }\right\}$. A quality‐of‐fit parameter is one of the two least‐squares objective functions,

(9) $$\chi^{2}=\sum_{i=1}^{N}{\left(R_{1,i}^{3\mathrm{TM}}-R_{1,i}^{\exp}\right)}^{2}\;\text{or}\;\chi^{2}=\sum_{i=1}^{N}{\left(\log_{10}R_{1,i}^{3\mathrm{TM}}-\log_{10}R_{1,i}^{\exp}\right)}^{2}$$

where $R_{1,i}^{\exp}$ is the i ^th^ of N experimental data points. The minimum $\chi^{2}$ is found for each combination of parameters $\left\{\tau_{l},\tau_{d},\tau_{b},x,N_{\sigma }\right\}$ so that the minimum $\chi^{2}$ is guaranteed to be found within the selected ranges. In practice, the logarithmic objective function is used when the largest relaxation rate is more than a factor of 10 larger than the smallest rate. See Table 2 for a list of parameters for each model that may be adjusted to secure the optimum fit.

The 3TM is applicable to a broad range of materials with fit parameters and derived parameters that have well‐defined physical meaning enabling improved insight and understanding of system properties. The component contributions to measured relaxation rates at each measurement frequency can be identified as demonstrated in Section 3. For sets of FFC NMR measurements that produce NMRD profiles as a function of a macroscopic property, changes in fit parameters may be linked to the property as illustrated in Section 3.4.

The primary shortcoming of the 3TM is that there is a ‘high barrier to entry’, that is, there are no straightforward equations that can be used for fitting. Relaxation rates are pre‐calculated and specialist software is required for fitting, either through an interface [54, 55] or through MATLAB software supplied via https://zenodo.org/records/18744873. General information and the scope of applications are provided on a dedicated website [56].

## 3 Applications of the 3TM

### 3.1 Grey Cement

Cementitious systems have proven a useful testing ground for FFC NMR models because the relaxation mechanism is well understood [1, 22, 23, 24, 57, 58]. Most cementitious material contains a distribution of paramagnetic iron (III) with high spin S=5/2. The HM model dominates. The NMRD profile $R_{1}^{\mathrm{HM}}\left(f\right)$ has contributions due to the motion of both bulk and bound water relative to fixed iron (III) in the solid as illustrated in Figure 1 and Equation (4). Here, 10 NMRD profiles presented by Badea et al. [58] for a hydrated grey cement at times from 15 min to 8 h after hydration (their Figure 3b at 15°C) are reanalysed using the 3TM. The 3TM fits include the HM model, a frequency‐independent offset $R_{1}^{\text{offset}}$ and a component $R_{1}^{\mathrm{aq}}\left(f\right)$ due to aqueous iron (III).

Aqueous paramagnetic ions can contribute significantly to the observed relaxation rate in some systems. Grey cement is one such example. The iron (III) impurities at the solid surfaces may desorb into the bulk water upon hydration. Faux et al. [7] conducted FFC NMR measurements of aqueous manganese (II), iron (III) and copper (II) chlorides where the scalar coupling contribution is known to be small. A Brownian shell model describing the random rotational motion of a spherical shell of uniform particle contributed to excellent fits across the full frequency range with physically justifiable numerical values of fit parameters. The parametrized fit for iron (III) is used here and referred to as the Fe (aq) model in Table 2. The magnitude of the Fe (aq) contribution is proportional to aqueous iron (III) density $N_{\mathrm{aq}}$.

**FIGURE 2:**
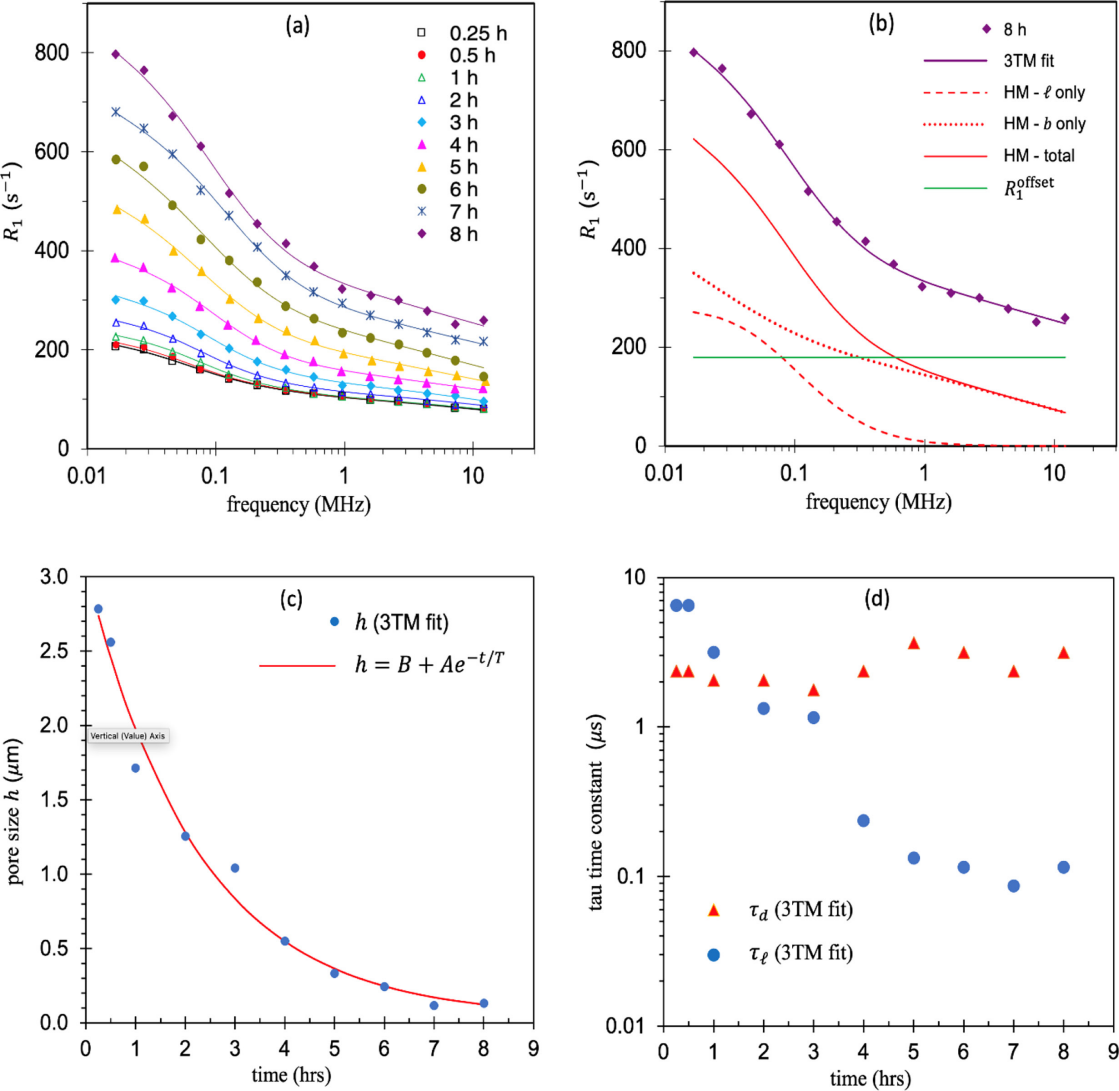
(a) 3TM fits from 15 min to 8 h post‐hydration for a grey cement sample at 15°C from [58]. (b) Two HM model contributions after 8 h of hydration. (c) Representative pore size as a function of hydration time with an exponential fit. (d) Decline of $\tau_{l}$ during hydration not matched by $\tau_{d}$.

The 10 NMRD profiles are reproduced in Figure 2a with the 3TM fits. The fit parameters are presented in Table 4.

**TABLE 4:** The 3TM best fit parameters are listed. $N_{\ell }$ was fixed at 66.6 spins/nm^3^ and not used as a fit parameter.

Sample	Grey cement, 15°C, Badea et al. [58]									
Models	HM + offset + Fe (aq)									
	Hydration	$\tau_{b}$	$\tau_{l}$	$\tau_{d}$	$\tau_{d}/\tau_{l}$	x	$N_{\sigma }\;$Fe^3+^	$N_{\mathrm{aq}}$	$R_{1}^{\text{offset}}$	h
	h	ps	μs	μs			10^−3^ Fe/nm^3^	10^−3^ Fe/nm^3^	s^−1^	μm
Badea‐1	0.25	27	6.5	2.4	0.37	0.19	6.8	0.27	42	2.8
Badea‐2	0.5	21	6.5	2.4	0.37	0.21	8.2	0.30	43	2.6
Badea‐3	1	21	3.2	2.1	0.65	0.32	8.4	0.30	43	1.7
Badea‐4	2	24	1.3	2.1	1.5	0.43	8.3	0.30	47	1.3
Badea‐5	3	32	1.2	1.8	1.5	0.52	8.6	0.30	47	1.0
Badea‐6	4	37	0.24	2.4	10	0.98	8.7	0.12	67	0.55
Badea‐7	5	65	0.13	3.7	27	1.62	7.3	0.00	78	0.33
Badea‐8	6	87	0.12	3.2	27	2.20	7.1	0.00	96	0.25
Badea‐9	7	87	0.09	2.4	27	4.61	6.4	0.00	150	0.12
Badea‐10	8	100	0.12	3.2	27	4.08	6.6	0.00	180	0.13

*Note:* The correlation times are restricted to 16 logarithmically spaced times per decade and may lead to identical values for different fits in some cases [55].

There are typically four pore water environments in hydrated cementitious material: capillary ( μm), interhydrate (10 + nm), gel (3–5 nm) and interlayer (~1.5 nm). The hydration reactions produce (principally) calcium silicate hydrate which consumes capillary water to produce smaller pore types. The FFC NMR experiment is unlikely to capture the water in the interlayer or gel pores due to the exceptionally short relaxation time of confined water [59].

The results after 8‐h hydration are presented in Figure 2b and reveal that, beyond about 1 MHz, the HM model contribution arises solely from interactions between iron (III) in the solid and bulk water close to the solid surfaces, labelled $R_{1}^{\mathrm{b\sigma}}$ in Equation (4), added to $R_{1}^{\text{offset}}\approx 190$ s^−1^. The water bound to the reaction products does not contribute. At the frequencies < 1 MHz, all three relaxation rates, $R_{1}^{\mathrm{b\sigma}}$, $R_{1}^{\mathrm{l\sigma}}$ and $R_{1}^{\text{offset}}$, contribute. $R_{1}^{\text{offset}}$ is associated with restricted diffusion of the pore water. See Table 4. This is because, as the hydration reactions progress, the hydrated pore spaces diminish in size and the aqueous ions, chiefly calcium and associated anions, become more concentrated. The free diffusion of pore water is restricted by the blocking effect of hydrated anions and cations and by the exchange with water bound to the aqueous ions. This reduces the water self‐diffusion coefficient resulting in increased pore water diffusion correlation time $\tau_{p}$ (see Equation 3 in Section 2.7) and increased relaxation rates [7].

The self‐diffusion coefficient of the pore water may be estimated from $R_{1}^{\text{offset}}$ using the HF model as described in Section 2.7. Pure water at room temperature has a self‐diffusion coefficient of $2.3\times 10^{-9}$ m^2^ s^−1^, which is frequency‐independent across the frequency range of a FFC NMR experiment and leads to $R_{1}^{\text{offset}}\approx 0.3$ s^−1^. The values of $R_{1}^{\text{offset}}$ seen here are orders of magnitude larger, the water dynamics are therefore orders of magnitude slower, and there is also a weak frequency dependence of the relaxation rate. Consequently, the self‐diffusion coefficients must be estimated for at a specific frequency. For consistency we choose to determine self‐diffusion coefficients at 20 MHz, which is a typical fixed field NMR frequency. An $R_{1}^{\text{offset}}$ of 42 s^−1^ (after 15 min of hydration) translates to $D_{\mathrm{HF}}\approx 1\times 10^{-11}$ m^2^ s^−1^ at 20 MHz. An $R_{1}^{\text{offset}}$ of 180 s^−1^ (after 8 h) measured at 20‐MHz approximates to $D_{\mathrm{HF}}\approx 1.2\times 10^{-12}$ m^2^ s^−1^.

Figure 2c presents the representative pore size h plotted as a function of hydration time and fit to an exponential function. This supplies a characteristic hydration time T=2.3 h. The rate of curing of cementitious material is linked to the ultimate strength of the product. The compressive strength of the final product is normally assessed after several months post hydration. If the hydration rate is slow (for instance because the cement has been exposed to a moist atmosphere for too long), the ultimate strength is compromised. Equally, if the curing rate is too fast, the final product also has poor properties. A series of FFC NMR experiments on different cement formulations coupled with 3TM analysis and strength assessment could, in principle, statistically link a curing rate with ultimate strength. This in turn would allow FFC NMR with 3TM analysis to estimate the ultimate strength of a product and do so after only a short period of hydration.

Figure 2d provides insight into the changes of surface chemistry due to chemical reactions that take place after hydration. The cement hydration chemistry is complex. The dominant reaction is of calcium silicate reacting with water to produce calcium silicate hydrate (CSH). Figure 2d shows $\tau_{l}$ declining by more than an order of magnitude during 8 h of hydration, demonstrating a significant weakening of the interaction strength between the solid and bound water. This is because the surface water is interacting with the CSH rather than the ionic solid and the interactions are weaker. What is surprising however is that the desorption time constant $\tau_{d}$ does not follow suit. The rate of water desorption from the surface to the bulk is normally linked to the mobility of surface water. Figure 2d sees little change in $\tau_{d}$. This may be associated with an increase in tortuosity of the surface which accompanies the hydration process.

A contribution due to Fe (aq) was added to the relaxation rate, arising due to the dissolution of iron (III) from the solid surfaces upon hydration. The Fe (aq) contribution is found to be small as expected, typically about 10 s^−1^ at its maximum, but improves the fits for hydration times to 4 h. See Table 4. The consistency of $N_{\mathrm{aq}}$ during the early stages of the hydration process suggests tentatively that the iron is being taken into the hydration products at the same rate as the water. From 5 h post hydration, the 3TM fits are not improved by the inclusion of a Fe (aq) component, implying complete absorption of the iron from solution. At present, there is no means of assessing these conclusions by alternative methods.

### 3.2 Plaster Paste

Plaster is calcium sulphate hemihydrate (CaSO_4_, ½H2O), or gypsum, which is hydrated to form a paste. FFC NMR measurements on a hydrated plaster free of paramagnetic ions were reported by Korb [1]. The water‐to‐plaster weight ratio was 0.8. The absence of paramagnetic spins makes the plaster a suitable test of the HH model and was used with the 3TM fitting software interface developed by Kogon and Faux [54, 55]. Here we use the 3TM fitting software to re‐visit the plaster paste to demonstrate that high‐quality 3TM fits can be obtained using different fitting strategies. In doing so, the sensitivity of some fit parameters to small changes in chosen model assumptions can be determined.

The two fit parameters tested are the relaxation rate offset $R_{1}^{\text{offset}}$ and surface water spin density $N_{l}$. Previously, $R_{1}^{\text{offset}}$ was set to zero, and $N_{l}$ was set to the bulk water spin density of 66.6 spins/nm^3^.

**FIGURE 3:**
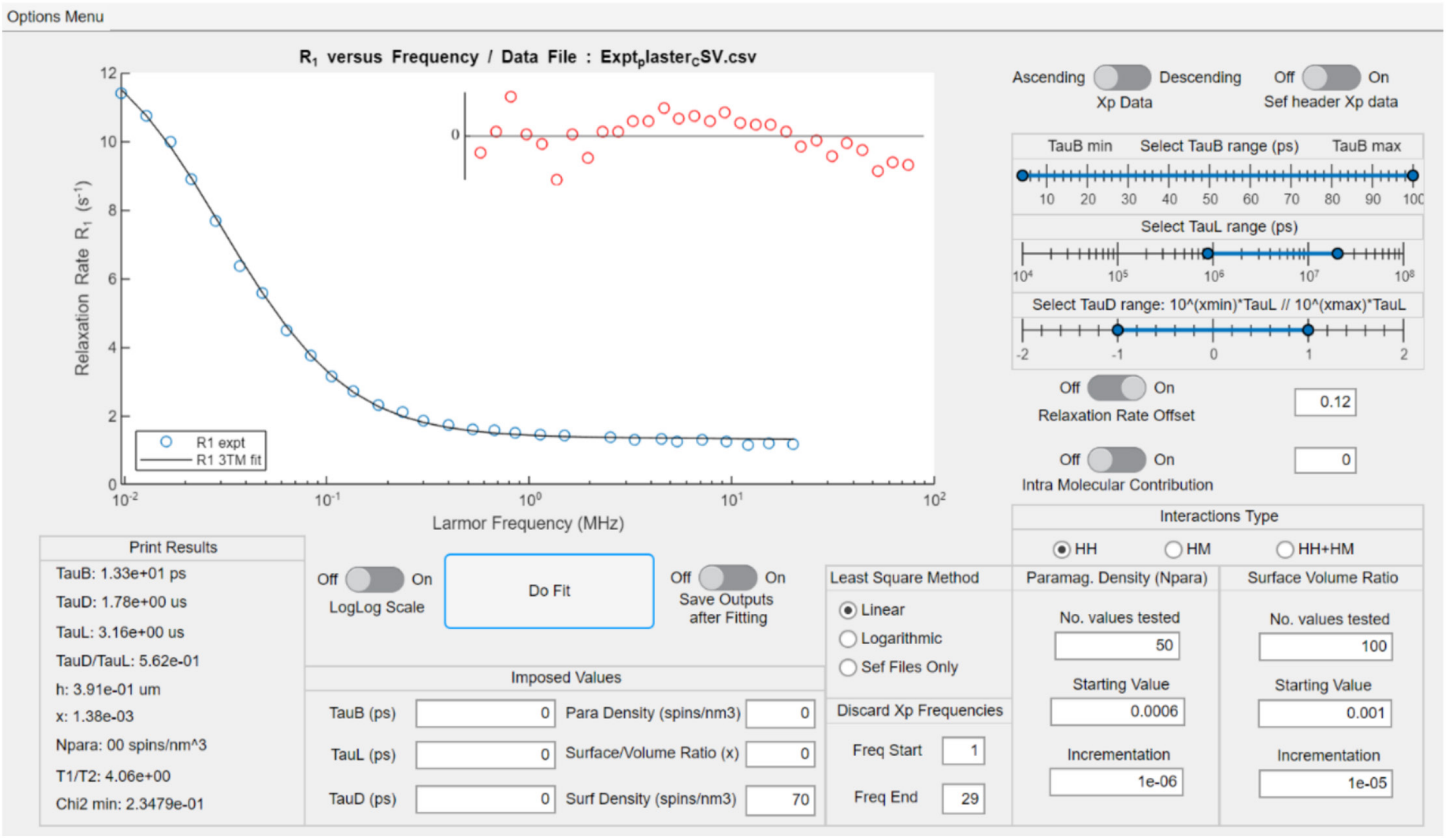
A screen grab of the 3TM fitting software interface showing an example fit.

Figure 3 presents a typical fit using the 3TM fitting software and Table 5 shows the outcome of three fits to the plaster paste. Fit‐1 assumed $R_{1}^{\text{offset}}=0$ and varied $N_{\ell }$ to secure the optimum fit. Fit‐2 varied $R_{1}^{\text{offset}}$ but fixed $N_{l}$ at 66.6 spins/nm^3^. Fit‐3 varied both $R_{1}^{\text{offset}}$ and $N_{l}$.

**TABLE 5:** The 3TM best fit parameters are listed for three fitting strategies.

Sample	Plaster paste, Korb (2011) [1]									
Models	HH (+ offset for Fit‐2 and Fit‐3)									
	Quality of fit $\chi^{2}$	$\tau_{b}$	$\tau_{l}$	$\tau_{d}$	$\tau_{d}/\tau_{l}$	$T_{1}/T_{2}$	$N_{l}$	$R_{1}^{\text{offset}}$	$R_{1}^{\mathrm{bb}}$	$D_{w}/D$
		ps	μs	μs		At 20 MHz	^1^H/nm^3^	s^−1^	s^−1^	
Fit‐1	0.319	15.5	3.16	1.78	0.56	3.6	72	0	0.83	3.0
Fit‐2	0.235	13.3	3.16	1.78	0.56	4.1	66.6	0.12	0.72	3.0
Fit‐3	0.235	13.3	3.16	1.78	0.56	4.1	68	0.12	0.72	3.0

*Note:* The offset is varied in Fit‐2 with a best fit value for $R_{1}^{\text{offset}}$of 0.12 s^−1^. In Fit‐3, both $N_{l}$ and $R_{1}^{\text{offset}}$ are varied with the optimum fit presented. The correlation times are restricted to 16 logarithmically spaced times per decade and may lead to identical values for different fits in some cases [55].

Fit‐2 and Fit‐3 show that inclusion of $R_{1}^{\text{offset}}$ significantly improves the quality of fit. Fit‐3 suggests that $R_{1}^{\text{offset}}=0.120\pm 0.005$ based on the range of values producing at most a 1% change in $\chi^{2}$. Fit‐2 shows that $\chi^{2}$ is insensitive to $N_{l}$. We estimate $N_{l}=68\pm 10$ spins/nm^3^. The numerical values of the remaining fit parameters are unchanged between Fit‐2 and Fit‐3.

All fits yield the ratio $T_{1}/T_{2}\approx 4$ at 20 MHz consistent with fixed field measurements for cementitious material [22]. The ratio for this specific plaster paste is not available. Note that the 3TM software allows a fixed $T_{1}/T_{2}$ ratio as a fit constraint if known.

$R_{1}^{\mathrm{bb}}$ is the relaxation rate for water bulk–bulk interactions calculated based on the dynamic time constant $\tau_{b}$. See Equation (5). $R_{1}^{\text{offset}}$ is normally associated with pore water bulk–bulk interactions which may or may not align with $R_{1}^{\mathrm{bb}}$. $\tau_{b}$ is most sensitive to the first four layers of water atop the bound water which molecular dynamics simulations find to be two to three times slower than pure water [60]. Here, $R_{1}^{\mathrm{bb}}\approx 0.12$ s^−1^ which converts (using the HF model) to a self‐diffusion coefficient $5.1\times 10^{-9}$ m^2^ s^−1^ which is a factor 2.2 larger than that for pure water at room temperature.

### 3.3 Hydrogel

Polysaccharide‐based hydrogels are three‐dimensional hydrophilic, polymeric networks, capable of imbibing large amounts of water (90%–98% of water content). They are of considerable interest in the pharmaceutical and medical fields, especially as slow‐release drug delivery applications, in the food industry and for household products such as diapers [61, 62, 63, 64, 65, 66]. Here, the FFC NMR measurements and 3TM analysis by Kogon et al. [67] of a hydrogel are reviewed.

The hydrogel was prepared as shown in the image as a single sample with a gradation of density. The sample was sliced into three sections for the FFC NMR experiment, labelled ‘far’, ‘middle’ and ‘near’. The NMR profile for each hydrogel slice was measured and interpreted using the 3TM interface. A summary of the fit outcomes is presented in Table 6.

**TABLE 6:** The 3TM best fit parameters are listed for each slice of the hydrogel.

Sample	Hydrogel, Kogon et al. (2023) [67]								
Models	HH								
**Region**	$\tau_{b}$	$\tau_{l}$	$\tau_{d}$	$\tau_{d}/\tau_{l}$	x	h	$N_{l}$	$R_{1}^{\text{offset}}$	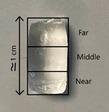
	**ps**	**μs**	**μs**			**nm**	^**1**^ **H/nm** ^**3**^	**s** ^**−1**^	
Far	6.4	0.56	1.53	2.73	17	9.6	20	0.182	
Middle	7.5	0.48	1.15	2.37	25	6.6	20	0.182	
Near	8.6	0.48	1.53	2.05	17	3.6	20	0.182	
			Sample average			6.6			
			Neutron scattering			7.5			

*Note:* The average pore spacing h from the 3TM and the neutron scattering result [67] are also shown. Note that reference [67] found h=22 spins/nm^3^ but failed to account for $N_{l}<N_{b}$. The values of h presented in the table use the correct Equation (8). The correlation times are restricted to 16 logarithmically spaced times per decade and may lead to identical values for different fits in some cases [55].

There were no detectable paramagnetic impurities and so the HH model was used. Trial fitting with $N_{l}$ set to the bulk water value of 66.6 spins/nm^3^ failed to produce satisfactory fits. Reducing $N_{l}$ enabled good fits to be obtained and $N_{l}$ was fixed at 20 spins/nm^3^. This was the first 3TM analysis in which it was necessary to reduce the surface proton spin density. Every subsequent 3TM fit on non‐ionic solid material necessitated a reduction of $N_{l}$ recognizing that water binds weakly to macromolecules, organic and soft materials compared with ionic solids.

The pore size, h, is a critical parameter for many hydrogel applications. The absorbency of diapers and the rate of release of slow‐release drug delivery systems are two examples whose success depends critically on hydrogel with optimized pore size, often referred to as ‘mesh’ size. Maire du Poset et al. applied small‐angle neutron scattering (SANS) to the pre‐cut sample on the PACE spectrometer at Laboratoire Léon Brillouin (CEA, Saclay, France) [67]. They found a mesh size of 7.5 nm. The similarity between the SANS result of 7.5 and 6.6 nm found using FFC NMR with 3TM analysis is excellent. SANS measurements may require access to a specialist facility booked months in advance. A FFC NMR experiment with 3TM analysis yields h in days and could provide a viable and cost‐effective alternative for determining a representative pore size. Moreover, agreement between h provided by the 3TM using Equation (8) and independent data from neutron scattering confirms that $N_{l}<N_{b}$ for the hydrogel and that 20 spins/nm^3^ is a reasonable measure of surface spin density.

**FIGURE 4:**
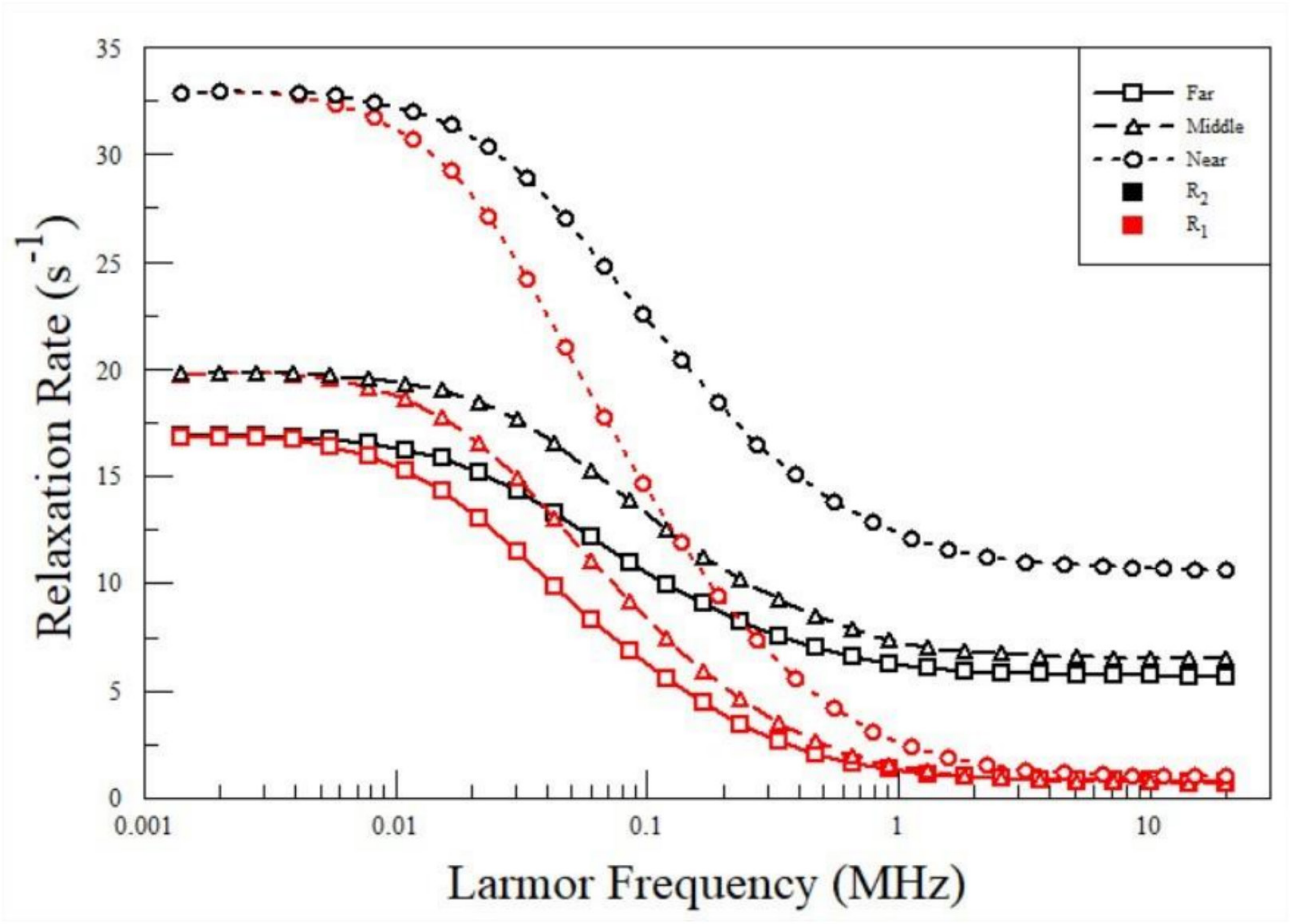
The figure is from reference [67]. The $R_{1}\left(f\right)$ relaxation rates (red lines) are the best fit curves to the experimental data from the ‘near’ (highest density), ‘middle’ (mid‐density) and ‘far’ (lowest density) hydrogel slices. The $R_{2}\left(f\right)$ relaxation rates (black lines) are calculated using the same values of the fit parameters obtained from the $R_{1}\left(f\right)$ best fit curves for each hydrogel slice. The symbols are not experimental data. The symbols identify the hydrogel slices as ‘near’ (circle) ‘middle’ (triangle) or ‘far’ (square) and are placed on each calculated NMRD profile at the same experimental frequencies used for each hydrogel slice.

The calculated relaxation rates $R_{1}\left(f\right)$ and $R_{2}\left(f\right)$ are presented in Figure 4 for each of the hydrogel slices. The $\;R_{1}\left(f\right)$ curves are the 3TM fits to the experimental data for each of the three slices and the $R_{2}\left(f\right)$ dispersions are generated using the same fit parameters. At the low frequencies, between 1 and 10 kHz, it is found that *R*_1_ and *R*_2_ are equal as expected. For f≳0.1 MHz it is seen that $R_{2}>R_{1}$ so that the ratio $T_{1}/T_{2}>1$ as observed experimentally. These results also supply the frequency dependence of the $T_{1}/T_{2}$ ratio.

### 3.4 Tumorous Tissue

**FIGURE 5:**
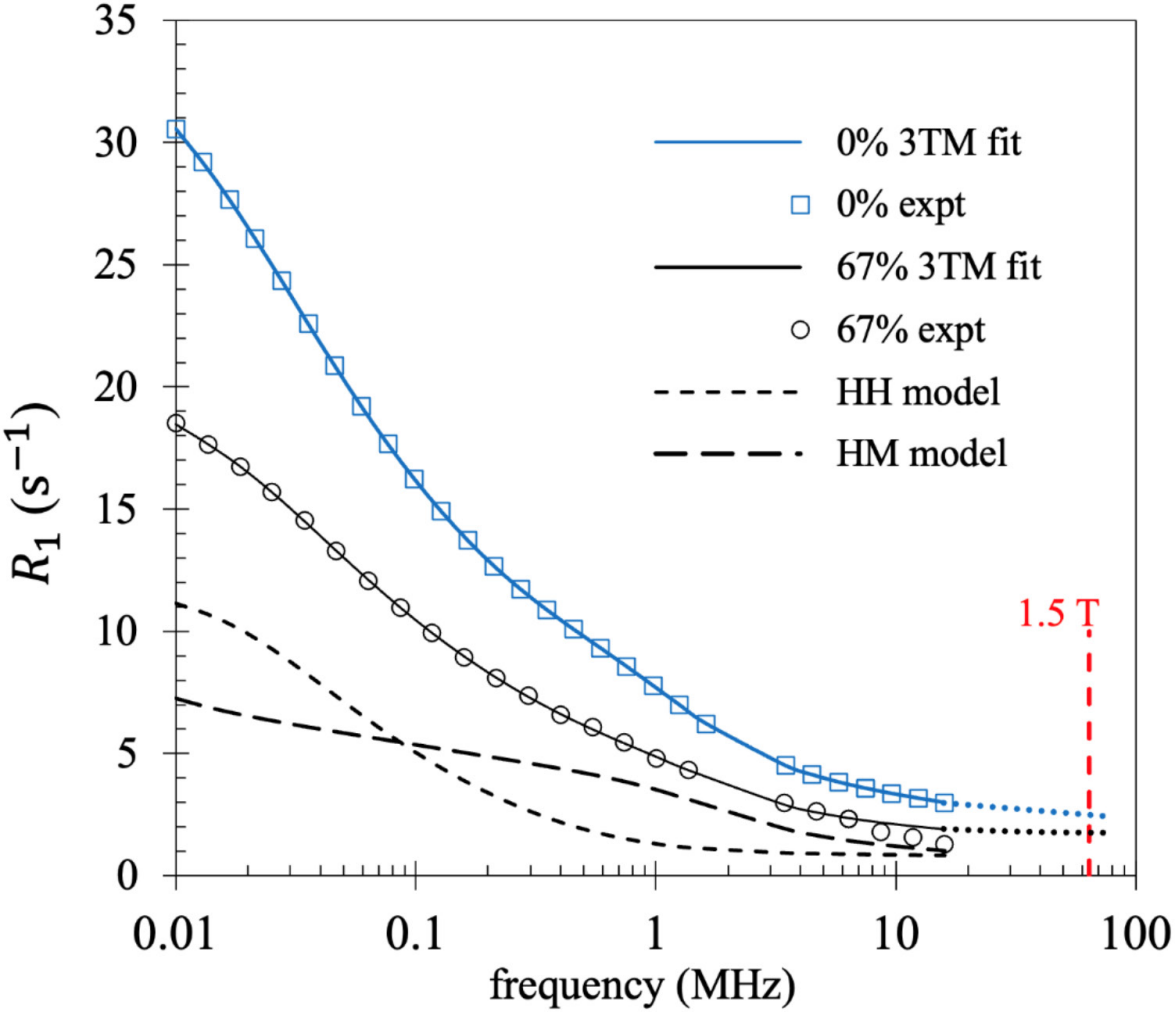
Examples of two 3TM fits (solid lines) to experimental data for the same mouse at tumour fraction c=0% (healthy) and c=67%. The HH model (short dashed line) and HM model (long dashed line) add the produce the c=67% fit. (Adapted from [68]).

We summarize the results of the first NMRD analysis to conclusively link a set of 3TM fit parameters to the change in a macroscopic property of a material [68]. Full details may be found in reference [68]. The 3TM analysis was undertaken on NMRD profiles from FFC NMR experiments by Ruggiero and co‐workers [69] with the datasets kindly supplied by Stelar s.r.l. In vivo FFC NMR measurements were conducted on 32 healthy and tumorous murine tissue samples from three different cell culture suppliers [69]. The motivation for the work is demonstrated in Figure 5, which compares NMRD profiles from just 2 of the 32 measurements: healthy tissue (tumour fraction c=0%) and tumorous tissue ( c=67%) for the same mouse. The two NMRD profiles converge at high fields with a typical MRI magnetic field of 1.5 T shown for reference. Healthy and tumorous tissue cannot be distinguished at 1.5 T. By contrast, at the lowest magnetic field, f=0.01 MHz, the FFC NMR measurements clearly distinguish the pathological tissue from the healthy tissue [69]. The fits to the NMRD profiles required both HH and HM models. The separate HM and HH model contributions for tumour fraction c=67% are presented in Figure 5.

A nucleus with a spin $I>\frac{1}{2}$ has a quadrupole moment which interacts with an electric field gradient resulting in quadrupolar resonances that may manifest as resonance peaks in the frequency range of an FFC NMR experiment. Quadrupolar peaks were first observed in protein systems [70] and attributed to water ^1^H–^14^N spin pairs. There are three resonance peaks in the range 0.5–3 MHz [8]. However, FFC NMR experiments do not have the frequency resolution to resolve the individual peaks and instead appears as a single broad peak centred at approximately 2 MHz. The murine tissue NMRD profiles are examples. It is important to account for the quadrupolar resonance for example by adding a Lorentzian function to the base model. Alternatively, as here, the data affected by the quadrupolar resonance may be removed from the fitting process as seen in Figure 5.

Both HM and HH models contribute to $R_{1}\left(f\right)$ at all frequencies. The NMRD profiles at low fields are sensitive to slow proton dynamics. The HM model is dominated by the interactions of slow‐moving bound water with fixed paramagnetic ions and the HH model is dominated by interactions between pairs of bound water protons. At the higher frequencies, $R_{1}\left(f\right)$ is most sensitive to the fast dynamics associated with bulk water. The HM and HH model contributions to $R_{1}\left(f\right)$ are dominated by interactions of bulk water with paramagnetic ions and surface water, respectively.

Each 3TM fit parameter was assessed to establish which, if any, showed a statistically significant change with tumour fraction. The 3TM fit parameters, including $R_{1}\left(f=0.01\;\mathrm{MHz}\right)$, are labelled $y_{i,j}$where the subscripts i,j refer to the j ^th^ dataset from the i ^th^ supplier ( i=1,2 or 3). Two models are then considered. Both models assume that y changes linearly with tumour fraction c and that the gradient β is independent of culture supplier. The two models are referred to as ‘parallel’ and ‘coincident’. The ‘parallel’ model allows the intercept $\alpha_{i}$ at c=0 (healthy cells) to be different for each culture supplier. The ‘coincident’ model sets the same intercept α in each case, so that [68]

(10) $$\text{parallel model}\;y_{i,j}=\alpha_{i}+\beta c_{i,j}+\epsilon_{i,j}$$

(11) $$\text{coincident model}\;y_{i,j}=\alpha +\beta c_{i,j}+\epsilon_{i,j}$$

where $\epsilon_{i,j}$ is a ‘noise’ term equal to the difference between the model and observation.

The results of the statistical analysis are presented in Table 7. The statistical significance of β is determined by the p value with p<0.05 considered significant. The adjusted coefficient of variation $R^{2}$ indicates the proportion of the variability in y accounted for by tumour fraction c. A biomarker is signalled by a small p‐value combined with a large $R^{2}$.

**TABLE 7:** The outcomes of the statistical analysis of the gradient β which identifies the fit parameters that act as tumour biomarkers.

Samples	Murine tissue samples [69]. Analysis by Faux et al. (2025) [68]			
Models	HM + HH			
Physical quantity	Model	Gradient β	p value	Adjusted $R^{2}$
$R_{1}\left(0.01\;\mathrm{MHz}\right)$	Coincident	−0.12	$4.3\times 10^{-12}$	0.796
$\tau_{l}$	None suitable	$-2.5\times 10^{-4}$	$4.7\times 10^{-6}$	0.069
$\tau_{d}$	Coincident	$-3.3\times 10^{-4}$	$1.6\times 10^{-4}$	0.363
$\tau_{b}$	Parallel	−0.24	$1.3\times 10^{-8}$	0.757
x	Parallel	$-3.1\times 10^{-5}$	$1.3\times 10^{-3}$	0.720
$N_{l}$	None suitable	$2.1\times 10^{-2}$	0.47	0.330
$N_{\sigma }$ Fe^3+^	None suitable	$-1.9\times 10^{-3}$	0.38	0.647

The three biomarkers are $R_{1}\left(0.01\;\mathrm{MHz}\right)$, $\tau_{b}$ and x. $R_{1}\left(0.01\;\mathrm{MHz}\right)$ is sensitive to tumour fraction with a sample containing 25% of tumour cells causing a decline of about 5 s^−1^. This result is independent of culture supplier. The increase in size of a tumour cell compared with a healthy cell arising from the net ingress of water explains why $\tau_{b}$ and x are biomarkers [68]. Both time constants $\tau_{l}$ and $\tau_{d}$ present statistically significant gradients, but $R^{2}$ is small. Nonetheless, the significant gradients suggest that structural changes of the surface of cell membranes may play a role in shaping the NMRD profiles. The spin densities $N_{\ell }$ and $N_{\sigma }$ present p≫0.05. This means that the gradients of both $N_{\ell }$ and $N_{\sigma }$ versus c (tumour fraction) are *not* significantly different from 0. Neither $N_{\ell }$ nor $N_{\sigma }$ depend on c and so there is no significant difference between healthy and tumorous tissues for these two fit parameters.

These results are promising and fully explored in reference [68]. Clearly further research is warranted to establish whether a single FFC NMR experiment across the frequency range 0.01–1.0 MHz from biopsy samples could provide separate estimates of tumour fraction from each identified biomarker.

### 3.5 Haemoglobin

Provisional results from the application of the 3TM to NMRD profiles from haemoglobin samples are presented. The NMRD profiles were published by Guevara et al. in 2022 [71] and kindly supplied by Manuel Guevara. Haemoglobin is the main content of red blood cells. The NMRD profiles were obtained from haemoglobin S (HbS) extracted from the red blood cells of two patients suffering from sickle cell anaemia and haemoglobin A (HbA) extracted from the red blood cells of five healthy patients. Each sample was then subjected to five or more repeat FFC NMR measurements. Full details of processing and experimental procedures may be found in reference [71]. The five or more NMRD profiles from each of the two HbS samples and five HbA samples were averaged. The averaged profiles were then re‐analysed using the 3TM.

**FIGURE 6:**
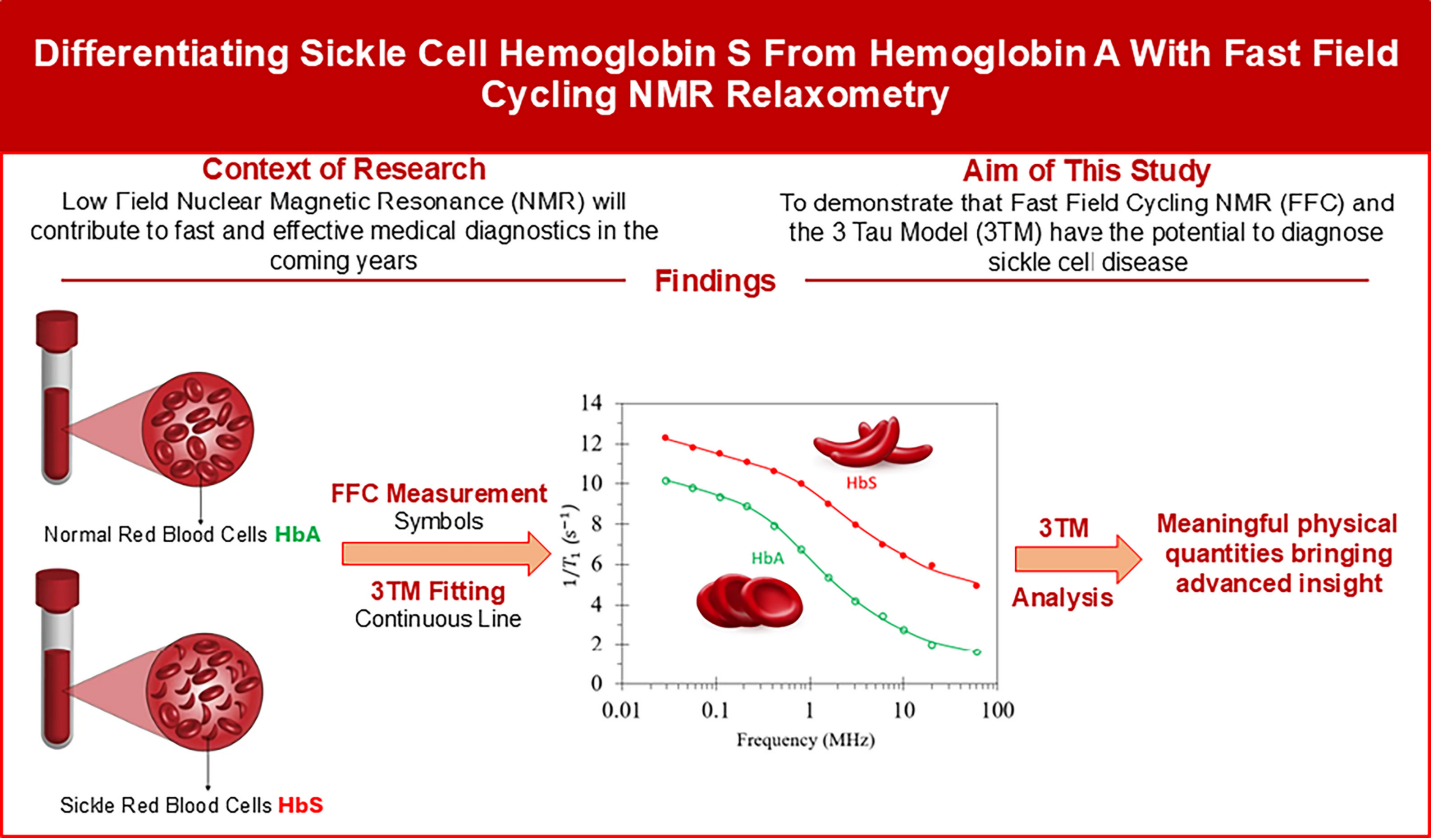
A summary of the outcome of 3TM fits to FFC NMR measurements of haemoglobin A from healthy red blood cells and haemoglobin S from blood cells with sickle cell anaemia. The plot illustrates the clear distinction between HbA and HbS.

The 3TM analysis required the HM, HH and SP models. The SP model provided a contribution less than 1 s^−1^, but the quality of fits was improved by its inclusion. The HH model made the dominant contribution to all seven NMRD profiles. The NMRD profiles and 3TM fits for datasets HbA‐1 and HbS‐1 are shown in the graphical abstract illustrated in Figure 6, which also highlights the motivation for the project.

The five HbA NMRD profiles yielded similar 3TM fit parameters. The mean and standard deviation are presented in Table 8. The two HbS profiles were also similar, and the mean and standard deviations of these fit parameters are also presented in Table 8.

**TABLE 8:** The means and standard deviations of fit parameters obtained from five HbA fits and two HbS fits are presented.

Samples	Haemoglobin A, haemoglobin S, Guevara et al. (2022) [71]						
Models	HM + HH + SP						
	$\tau_{b}$	$\tau_{l}$	$\tau_{d}$	$\tau_{d}/\tau_{l}$	$N_{SP}$	$N_{l}$	$N_{\sigma }\;$
	ps	μs	μs		^1^H/nm^3^	^1^H/nm^3^	10^−5^ Fe^3+^/nm^3^
HbA	52 ± 21	0.95 ± 0.10	0.033 ± 0.002	0.035 ± 0.004	19 ± 2	18 ± 7	9.4 ± 1.5
HbS	93 ± 7	0.49 ± 0.07	0.017 ± 0.001	0.035 ± 0.003	29 ± 3	17 ± 3	5.1 ± 0.8

The surface water dynamic correlation times $\tau_{l}$ and $\tau_{d}$ for HbA are approximately twice the values for HbS. There is therefore a significant difference of bound water interactions between HbA and HbS signalling a difference in surface chemistry. It is interesting that the ratio $\tau_{d}/\tau_{l}$ is the same for both HbS and HbA, and the small value of the ratio indicates that water desorption is significantly more likely than migration across the haemoglobin surface. There are also indications that the proton density of the haemoglobin, $N_{\mathrm{SP}}$, is larger in HbS samples compared to HbA possibly associated with polymer fragments in the HbS solution. The polymers and polymer fragments in the HbS solution cause obstructions and hydration effects and explain the significantly larger $\tau_{b}$ in HbS.

Finally, the paramagnetic spin density, $N_{\sigma }$, is associated with heme iron and its value in HbS is found to be about half that of the HbA samples. This result may be an indication that the heme iron is closer to the bound water in HbS samples due to polymerization, manifesting as a smaller value of $N_{\sigma }$ as observed.

These results suggest that FFC NMR with 3TM fitting has the potential to identify sickle cell anaemia in processed blood taken from patients.

## 4 Final Remarks

The NMRD profiles obtained from FFC NMR experimentation contain a rich source of information on the dynamics of proton spins in hydrated materials. Extracting this information poses a significant challenge. We have demonstrated that, for a broad range of material systems, the 3TM can not only provide realistic dynamic parameters but can also assess pore‐size changes, surface chemistry, paramagnetic ion density and, aided by the HF model, can estimate the self‐diffusion coefficient of the pore water. Moreover, we have provided evidence that FFC NMR can now connect with end users through the identification of tumour biomarkers, the potential to link cement curing rates and surface chemistry to macroscopic material properties, and to distinguish between healthy blood and blood with sickle cell anaemia.

Future developments of the 3TM approach are planned. The software has been adapted to fit to the spin–lattice relaxation rate in the rotating frame, $R_{1\rho }=1/T_{1\rho }$, with some provision fits completed to water/ice in silica sol–gel pores. The relaxation rate dispersion is as a function of the radio frequency field at a field applied magnetic field and accesses the frequency range 0.001–0.1 MHz. This is important because it makes the benefits of the 3TM analysis accessible to fixed field NMR relaxometry laboratories.

Future versions of the 3TM software will include an automated uncertainty analysis for each fitting. The present calculations are undertaken in four or five parameter space with the best fit (minimum $\chi^{2}$) guaranteed. The user may specify a variation from the minimum $\chi^{2}$ (2% say) and the software will return the variation of each fit parameter satisfying the criterion.

## Funding

The 3TM model and software was funded by the European Union Horizon 2020 Research and Innovation Programme under the Marie Skłodowska Curie Innovative Training Networks programme (grant agreement No. 764691).

## Conflicts of Interest

The authors declare no conflicts of interest.

## Data Availability

The data that support the findings of this study are available from the corresponding author upon reasonable request.

## References

[1] J.‐P. Korb , “Nuclear Magnetic Relaxation of Liquids in Porous Media,” New Journal of Physics 13 (2011): 035016.

[2] R. M. Steele , J.‐P. Korb , G. Ferrante , and S. Bubici , “New Applications and Perspectives of Fast Field Cycling NMR Relaxometry,” Magnetic Resonance in Chemistry 54 (2016): 502–509. 25855084 10.1002/mrc.4220

[3] P. Conte , Annual Reports on NMR Spectroscopy, vol. 2021, (Elsevier, 2021), 104–141.

[4] M. Bödenler , L. de Rochefort , P. J. Ross , et al., “Comparison of Fast Field‐Cycling Magnetic Resonance Imaging Methods and Future Perspectives,” Molecular Physics 117, no. 7–8 (2019): 832–848.

[5] L.‐P. Hwang and J. H. Freed , “Dynamic Effects of Pair Correlation Functions on Spin Relaxation by Translational Diffusion in Liquids,” Journal of Chemical Physics 63 (1975): 4017–4025.

[6] Y. Ayant , E. Belorizky , J. Aluzon , and J. Gallice , “Calcul des Densités Spectrales Résultant d'un Mouvement Aléatoire de Translation en Relaxation par Interaction Dipolaire Magnétique Dans les Liquides,” Journal de Physique 36 (1975): 991–1004.

[7] D. A. Faux , Ö. Istok , A. A. Rahaman , P. J. McDonald , D. Brougham , and E. McKiernan , “Nuclear Spin Relaxation in Aqueous Paramagnetic Ion Solutions,” Physical Review E 107 (2023): 054605. 37328976 10.1103/PhysRevE.107.054605

[8] E. P. Sunde and B. Halle , “Mechanism of ^1^H–^14^N Cross‐Relaxation in Immobilized Proteins,” Journal of Magnetic Resonance 203 (2010): 257–273. 20163976 10.1016/j.jmr.2010.01.008

[9] D. A. Faux , P. J. McDonald , and N. C. Howlett , “Nuclear‐Magnetic‐Resonance Relaxation due to the Translational Diffusion of Fluid Confined to Quasi‐Two‐Dimensional Pores,” Physical Review E 95 (2017): 033116. 28415296 10.1103/PhysRevE.95.033116

[10] D. A. Faux and P. J. McDonald , “Explicit Calculation of Nuclear‐Magnetic‐Resonance Relaxation Rates in Small Pores to Elucidate Molecular‐Scale Fluid Dynamics,” Physical Review E 95 (2017): 033117. 28415374 10.1103/PhysRevE.95.033117

[11] A. Abragam , The Principles of Nuclear Magnetism (Clarendon, 1961).

[12] N. Bloembergen , E. M. Purcell , and R. V. Pound , “Relaxation Effects in Nuclear Magnetic Resonance Absorption,” Physics Review 73, no. 7 (1948): 679–712.

[13] I. Solomon , “Relaxation Processes in a System of Two Spins,” Physics Review 99 (1955): 559–565.

[14] I. Solomon and N. Bloembergen , “Nuclear Magnetic Interactions in the HF Molecule,” Journal of Chemical Physics 25 (1956): 261–266.

[15] G. Laukien and J. Schlüter , “Impulstechnische Messungen der Spin–Gitter‐ und der Spin–Spin–Relaxationszeiten von Protonen in wäßrigen Lösungen paramagnetischer Ionen,” Zeitschrift für Physik 146 (1956): 113.

[16] L. Morgan and A. Nolle , “Proton Spin Relaxation in Aqueous Solutions of Paramagnetic Ions. II. Cr^+++^, Mn^++^, Ni^++^, Cu^++^, and Gd^+++^ ,” Journal of Chemical Physics 31 (1959): 365–368.

[17] N. Bloembergen and L. Morgan , “Proton Relaxation Times in Paramagnetic Solutions. Effects of Electron Spin Relaxation,” Journal of Chemical Physics 34 (1961): 842.

[18] J.‐P. Korb , S. Xu , and J. Jonas , “Confinement Effects on Dipolar Relaxation by Translational Dynamics of Liquids in Porous Silica Glasses,” Journal of Chemical Physics 98 (1993): 2411–2422.

[19] J.‐P. Korb , M. Whaley‐Hodges , and R. G. Bryant , “Theoretical Analysis of Field‐Cycling Magnetic Relaxation of Liquids in Micro‐Heterogeneous Systems,” Physical Review E 56 (1997): 1934.

[20] S. Godefroy , J.‐P. Korb , M. Fleury , and R. G. Bryant , “Surface Nuclear Magnetic Relaxation and Dynamics of Water and Oil in Macroporous Media,” Physical Review E 64 (2001): 021605. 10.1103/PhysRevE.64.021605 11497601

[21] F. Barberon , J.‐P. Korb , D. Petit , V. Morin , and E. Bermejo , “Probing the Surface Area of a Cement‐Based Material by Nuclear Magnetic Relaxation Dispersion,” Physical Review Letters 90 (2003): 116103. 12688946 10.1103/PhysRevLett.90.116103

[22] P. J. McDonald , J.‐P. Korb , J. Mitchell , and L. Monteilhet , “Surface Relaxation and Chemical Exchange in Hydrating Cement Pastes: A Two‐Dimensional NMR Relaxation Study,” Physical Review E 72 (2005): 011409. 10.1103/PhysRevE.72.011409 16089963

[23] A. Plassais , M.‐P. Pomiés , N. Lequeux , et al., “Microstructure Evolution of Hydrated Cement Pastes,” Physical Review E 72 (2005): 041401. 10.1103/PhysRevE.72.041401 16383375

[24] L. Monteilhet , J.‐P. Korb , J. Mitchell , and P. J. McDonald , “Observation of Exchange of Micropore Water in Cement Pastes by Two‐Dimensional T2−T2 Nuclear Magnetic Resonance Relaxometry,” Physical Review E 74 (2006): 061404. 10.1103/PhysRevE.74.061404 17280070

[25] J.‐P. Korb , B. Nicot , A. Louis‐Joseph , S. Bubici , and G. Ferrante , “Dynamics and Wettability of Oil and Water in Oil Shales,” Journal of Physical Chemistry C 118 (2014): 23212–23218.

[26] V. P. Denisov and B. Halle , “Protein Hydration Dynamics in Aqueous Solution,” Faraday Discussions 103 (1996): 227. 10.1039/fd9960300227 9136639

[27] V. P. Denisov , J. Peters , H. D. Hōrlein , and B. Halle , “Using Buried Water Molecules to Explore the Energy Landscape of Proteins,” Nature Structural Biology 3 (1996): 505–509. 8646535 10.1038/nsb0696-505

[28] K. Venu , V. P. Denisov , and B. Halle , “Water ^1^H Magnetic Relaxation Dispersion in Protein Solutions. A Quantitative Assessment of Internal Hydration, Proton Exchange, and Cross‐Relaxation,” Journal of the American Chemical Society 119 (1997): 3122–3134.

[29] B. Halle and V. P. Denisov , “Magnetic Relaxation Dispersion Studiesof Biomolecular Solutions,” Methods in Enzymology 338 (2002): 178–201. 10.1016/s0076-6879(02)38220-x 11460548

[30] B. Halle , “Protein Hydration Dynamics in Solution: A Critical Survey,” Philosophical Transactions of the Royal Society of London. Series B: Biological Sciences 359, no. 1448 (2004): 1207–1224. 15306377 10.1098/rstb.2004.1499 PMC1693401

[31] K. Modig , E. Liepinsh , G. Otting , and B. Halle , “Dynamics of Protein and Peptide Hydration,” Journal of the American Chemical Society 126 (2004): 102–114. 14709075 10.1021/ja038325d

[32] M. Lores and C. Cabal , “Proton Magnetic Relaxation Process During the Polymerization of Hemoglobin S,” Applied Magnetic Resonance 28, no. 1 (2005): 79–84.

[33] Y. Cabrales , M. A. Lores‐Guevara , and Y. Machado , “Deuterium Magnetic Relaxation Process During the Polymerization of Hemoglobin S,” Applied Magnetic Resonance A33 (2008): 207.

[34] M. A. Lores‐Guevara , C. A. Cabal‐Mirabal , R. N. Müller , S. Laurent , F. Tamayo‐Delgado , and J. C. García‐Naranjo , “Proton MRD Profile Analysis in Intracellular Hemoglobin Solutions: A Three Sites Exchange Model Approach,” Applied Magnetic Resonance A53 (2022): 387.

[35] D. Kruk , A. Kasparek , E. Masiewicz , K. Kolodziejski , R. Cybulski , and B. Nowak , “Water Dynamics in Highly Concentrated Protein Systems—Insight From Nuclear Magnetic Resonance Relaxometry,” International Journal of Molecular Sciences 24, no. 4 (2023): 4093. 36835511 10.3390/ijms24044093 PMC9963861

[36] F. V. Chávez and B. Halle , “Molecular Basis of Water Proton Relaxation in Gels and Tissue,” Magnetic Resonance in Medicine 56, no. 1 (2006): 73–81. 16732591 10.1002/mrm.20912

[37] B. Halle , “Spin Dynamics of Exchanging Quadrupolar Nuclei in Locally Anisotropic Systems,” Progress in Nuclear Magnetic Resonance Spectroscopy 28, no. 2 (1996): 137–159.

[38] L. Kruse , A. M. Chiramel Tony , D. Paschek , P. Stange , R. Ludwig , and A. Strate , “Translational Dynamics of Cations and Anions in Ionic Liquids from NMR Field Cycling Relaxometry: Highlighting the Importance of Heteronuclear Contributions,” Journal of Physical Chemistry Letters 15, no. 41 (2024): 10410–10415. 39387540 10.1021/acs.jpclett.4c02245

[39] L. Kruse , T. van Alphen , J. Busch , D. Paschek , R. Ludwig , and A. Strate , “Beyond Isotropic Reorientation: Probing Anisotropic and Internal Motions in Ionic Liquids With Fast Field Cycling NMR Relaxometry and MD Cimulations,” Physical Chemistry Chemical Physics 27, no. 21 (2025): 10927–10938. 40195741 10.1039/d5cp00582e

[40] C. A. Sholl , “Nuclear Spin Relaxation by Translational Diffusion in Liquids and Solids: High‐ and Low‐Frequency Limits,” Journal of Physics C 14 (1981): 447–464.

[41] B. Halle , H. Jóhannesson , and K. Venu , “Model‐Free Analysis of Stretched Relaxation Dispersions,” Journal of Magnetic Resonance 135 (1998): 1–13. 9799667 10.1006/jmre.1998.1534

[42] P. J. Sebastião , “The art of model fitting to experimental results,” European Journal of Physics 35, no. 1 (2014): 015017.

[43] T. R. Lindstrom and S. H. Koenig , “Magnetic‐Field‐Dependent Water Proton Spin‐Lattice Relaxation Rates of Hemoglobin Solutions and Whole Blood,” Journal of Magnetic Resonance (1969) 15, no. 2 (1974): 344–353.

[44] K. Hallenga and S. H. Koenig , “Protein Rotational Relaxation as Studied by Solvent Proton and Deuteron Magnetic Relaxation,” Biochemist 15, no. 19 (1976): 4255–4264. 10.1021/bi00664a019 963035

[45] P. Lo Meo , S. Terranova , A. Di Vincenzo , D. Chillura Martino , and P. Conte , “Heuristic Algorithm for the Analysis of Fast Field Cycling (FFC) NMR Dispersion Curves,” Analytical Chemistry 93 (2021): 8553–8558. 34102062 10.1021/acs.analchem.1c01264

[46] G. Landi , G. V. Spinelli , F. Zama , et al., “An Automatic L1‐Based Regularization Method for the Analysis of FFC Dispersion Profiles With Quadrupolar Peaks,” Applied Mathematics and Computation 444 (2023): 127809.

[47] V. Bortolotti , P. Conte , G. Landi , et al., “Robust Algorithms for the Analysis of Fast‐Field‐Cycling Nuclear Magnetic Resonance Dispersion Curves,” Computer 13 (2024): 129.

[48] L. D. Favro , “Theory of the Rotational Brownian Motion of a Free Rigid Body,” Physics Review 119 (1960): 53–62.

[49] C. Calero , J. Martí , and E. Guárdia , “ ^1^H Nuclear Spin Relaxation of Liquid Water From Molecular Dynamics Simulations,” Journal of Physical Chemistry. B 119 (2015): 1966. 25584483 10.1021/jp510013q

[50] D. A. Faux , A. A. Rahaman , and P. J. McDonald , “Water as a Lévy Rotor,” Physical Review Letters 127 (2021): 256001. 35029422 10.1103/PhysRevLett.127.256001

[51] D. Paschek , J. Busch , A. M. Chiramel Tony , et al., “When Theory Meets Experiment: What Does It Take To Accurately Predict ^1^H NMR Dipolar Relaxation Rates in Neat Liquid Water From Theory?,” Journal of Chemical Physics 162 (2025): 5. 10.1063/5.0249826 39898566

[52] F. Winter and R. Kimmich , “Spin Lattice Relaxation of Dipole Nuclei (I = 1/2) Coupled to Quadrupole Nuclei (S = 1),” Molecular Physics 45 (1982): 33–49.

[53] M. M. Rusu , D. A. Faux , and I. Ardelean , “Monitoring the Effect of Calcium Nitrate on the Induction Period of Cement Hydration via Low‐Field NMR Relaxometry,” Molecules 28, no. 2 (2023): 476. 36677533 10.3390/molecules28020476 PMC9862773

[54] K. Krynicki , “Proton spin‐lattice relaxation in pure water between 0°C and 100°C,” Physica 32, no. 1 (1966): 167–178.

[55] R. A. Kogon and D. A. Faux , 3TM Fitting Software for Fast Field Cycling NMR Dispersion Data (Zenodo, 2021), 10.5281/zenodo.5774107.

[56] R. A. Kogon and D. A. Faux , “3TM: Software for the 3‐Tau Model,” SoftwareX 17 (2022): 100979.

[57] D. A. Faux and R. A. Kogon , “Examples of Applications of the 3‐Tau Model and Access to the Latest Software May Be Found on the DR Relaxo Scientific Consultancy Website at dr‐relaxo.com”.

[58] J. P. Korb , L. Monteilhet , P. J. McDonald , and J. Mitchell , “Microstructure and Texture of Hydrated Cement‐Based Materials: A Proton Field Cycling Relaxometry Approach,” Cement and Concrete Research 37 (2007): 295–302.

[59] C. Badea , A. Pop , C. Mattea , S. Stapf , and I. Ardelean , “The Effect of Curing Temperature on Early Hydration of Gray Cement Via Fast Field Cycling‐NMR Relaxometry,” Applied Magnetic Resonance 45, no. 12 (2014): 1299–1309.

[60] D. A. Faux and P. J. McDonald , “Nuclear‐Magnetic‐Resonance Relaxation Rates for Fluid Confined to Closed, Channel, or Planar Pores,” Physical Review E 98, no. 6 (2018): 063110.

[61] D. A. Faux , S.‐H. P. P. Cachia , P. J. McDonald , N. C. Howlett , J. S. Bhatt , and S. V. Churakov , “Model for the Interpretation of Nuclear Magnetic Resonance Relaxometry of Hydrated Porous Silicate Materials,” Physical Review E 91 (2015): 032311. 10.1103/PhysRevE.91.032311 25871114

[62] R. Kimmich , Principles of Soft‐Matter Dynamics (Springer, 2012).

[63] C.‐C. Lin and A. T. Metters , “Hydrogels in Controlled Release Formulations: Network Design and Mathematical Modeling,” Advanced Drug Delivery Reviews 58, no. 12‐13 (2006): 1379–1408. 17081649 10.1016/j.addr.2006.09.004

[64] T. Coviello , P. Matricardi , C. Marianecci , and F. Alhaique , “Polysaccharide Hydrogels for Modified Release Formulations,” Journal of Controlled Release 119, no. 1 (2007): 5–24. 17382422 10.1016/j.jconrel.2007.01.004

[65] J. Li and D. J. Mooney , “Designing Hydrogels for Controlled Drug Delivery,” Nature Reviews Materials 1, no. 12 (2016): 1. 10.1038/natrevmats.2016.71 PMC5898614 29657852

[66] A. Heumann , A. Assifaoui , D. da Silva Barreira , et al., “Intestinal Release of Biofilm‐Like Microcolonies Encased in Calcium‐Pectinate Beads Increases Probiotic Properties of *Lacticaseibacillus paracasei* ,” Biofilms and Microbiomes 6, no. 1 (2020): 44. 33116127 10.1038/s41522-020-00159-3 PMC7595111

[67] A. M. du Poset , M. Börjesson , C. Rameau , et al., “Controlled Loading and Release of Beta‐Lactoglobulin in Calcium‐Polygalacturonate Hydrogels,” Biomacromolecules 21, no. 4 (2020): 1417. 32109357 10.1021/acs.biomac.9b01722

[68] R. A. Kogon , D. A. Faux , A. Assifaoui , and P. Bodart , “Advanced Insight on the Water Dynamics of Anisotropic Hydrogels by Field‐Cycling Nuclear Magnetic Resonance: Application of 3‐Tau Model,” Carbohydrate Polymers 314 (2023): 120922. 37173021 10.1016/j.carbpol.2023.120922

[69] D. A. Faux , R. A. Kogon , and J. Godolphin , “Understanding Water Properties in Tumorous Murine Cells Using Field‐Cycling NMR Relaxometry,” Scientific Reports 16 (2026): 75, 10.1038/s41598-025-28860-3. PMC12764788 41453974

[70] M. R. Ruggiero , S. Baroni , S. Pezzana , G. Ferrante , S. Geninatti Crich , and S. Aime , “Evidence for the Role of Intracellular Water Lifetime as a Tumour Biomarker Obtained by In Vivo Field‐Cycling Relaxometry,” Angewandte Chemie 130, no. 25 (2018): 7590–7594. 10.1002/anie.201713318 PMC6175164 29575414

[71] M. A. Guevara , C. A. Mirabal , R. N. Muller , S. Laurent , F. T. Delgado , and J. C. G. Naranjo , “Proton MRD Profile Analysis in Intracellular Hemoglobin Solutions: A Three Sites Exchange Model Approach,” Applied Magnetic Resonance 53, no. 2 (2022): 387–399.

